# Predictors of ovarian reserve in young women with breast cancer

**DOI:** 10.1038/sj.bjc.6603814

**Published:** 2007-05-29

**Authors:** K Lutchman Singh, S Muttukrishna, R C Stein, H H McGarrigle, A Patel, B Parikh, N P Groome, M C Davies, R Chatterjee

**Affiliations:** 1 Department of Obstetrics and Gynaecology, Royal Free and University College London Medical School, 86-96 Chenies Mews, London WC1E 6HX, UK; 2 Department of Oncology, Royal Free and University College London Medical School, 91 Riding House Street, London W1W 7BS, UK; 3 Centre for Proteins and Peptides, School of Biological and Molecular Sciences, Oxford Brookes University, Headington, Oxford OX3 0BP, UK

**Keywords:** ovarian reserve, breast cancer, chemotherapy, fertility and premature ovarian failure

## Abstract

Ovarian reserve can be diminished following treatment for breast cancer. This study evaluated biochemical and biophysical parameters of ovarian reserve in these patients. Biochemical and biophysical tests of ovarian reserve were performed simultaneously in young (age 22–42 years), regularly menstruating women with breast cancer (*n*=22) and age-matched controls (*n*=24). All tests were performed before (baseline) and after transient ovarian stimulation in the early follicular phase. Patients were recruited both before and after completion of chemotherapy, with some patients being followed up prospectively. Serum samples were analysed for follicle-stimulating hormone (FSH), luteinising hormone (LH), oestradiol (E_2_), inhibins A and B, and antimullerian hormone (AMH). Biophysical (ultrasound) tests included ovarian volume, antral follicle count (AFC), ovarian stromal blood flow and uterine dimensions. Significant differences were revealed (when compared with the controls) for basal FSH (11.32±1.48 *vs* 6.62±0.42 mIU ml^−1^, *P*<0.001), basal AMH (0.95±0.34 *vs* 7.89±1.62 ng ml^−1^, *P*<0.001) and basal inhibin B (19.24±4.56 *vs* 83.61±13.45 pg ml^−1^, *P*<0.001). Following transient ovarian stimulation, there were significant differences in the increment change (Δ) for inhibin B (3.02±2.3 *vs* 96.82±16.38 pg ml^−1^, *P*<0.001) and E_2_ (107.8±23.95 *vs* 283.2±40.34 pg ml^−1^, *P*<0.01). AFC was the only biophysical parameter that was significantly different between patients and the controls (7.80±0.85 *vs* 16.77±1.11, *P*<0.001). Basal and stimulated biochemical (serum AMH, FSH, inhibin B and E_2_) and biophysical (AFC) tests may be potential markers of ovarian reserve in young women with breast cancer.

As the incidence of breast cancer has progressively increased, survival rates have simultaneously improved, owing to improvements in early detection and adjuvant chemotherapy (Jatoi and Miller, 2003). Young women with breast cancer are considered high risk (Goldhirsch *et al*, 2001), and are likely to receive chemotherapy, as the magnitude of benefit seems to be enhanced (Gradishar, 2003). As a result, these women are likely to suffer ovarian damage from chemotherapy, which can have a profound effect on their quality of life.

At present, it is impossible to predict the lifespan of the chemotherapeutically damaged ovary. Ovarian reserve testing has become established in the fertility setting where it is used to predict outcome in assisted reproduction (Bukulmez and Arici, 2004). These tests have the potential to estimate the reproductive lifespan of the ovaries, which would allow an accurate estimation of fertility status and the risk of premature ovarian failure.

There is a need for such tests to be validated in patients with breast cancer, so that the information needs of these patients can be met, and appropriate measures taken to improve their fecundity and overall quality of life.

The aim of this study was to evaluate ovarian function in young women treated by multi-agent, cyclophosphamide-based chemotherapy for breast cancer by using clinical, biochemical and biophysical parameters. This was based on the hypothesis that cytotoxic drugs used to treat premenopausal patients with breast cancer can cause ovarian damage resulting in diminished ovarian reserve.

## PATIENTS AND METHODS

In this pilot study, pre-menopausal recipients of chemotherapy for breast cancer were analysed in two groups – a longitudinal arm (group 1) and a cross-sectional arm (group 2). As all patients were recruited from a single centre, the chemotherapy regimens employed were standardised. There were two predominant regimens, both cyclophosphamide-based. The first was E-CMF, which consisted of four cycles of epirubicin (E), followed by four cycles of Cyclophosphamide (C), methotrexate (M) and 5-fluorouracil (F). The second was FEC, which consisted of six cycles of 5-fluorouracil, epirubicin and cyclophosphamide. Some patients received taxanes (docetaxel or paclitaxel), usually as part of a therapeutic trial (TANGO). The cumulative dosages received by patients in the longitudinal analysis are displayed in Table 1.

In group 1, patients were offered ovarian reserve testing before receiving chemotherapy. The same patients were then followed up longitudinally by performing further testing immediately following chemotherapy. In group 2, patients were tested for ovarian reserve before chemotherapy (some patients were also part of the longitudinal study) and after chemotherapy (once regular menstrual cycles had resumed). Patients with oestrogen-sensitive cancer were recruited provided they had not taken tamoxifen or gonadotrophin releasing hormone analogue (GnRHa) (goserelin or leuprolin) for at least the preceding 12 weeks.

Controls were age matched, had no medical illness, had proven fertility (defined by virtue of having at least one childbirth previously) and had a normal menstrual history. Those on oral contraception were asked to discontinue it and use alternative (barrier) contraception for at least 8 weeks before testing.

Informed consent was obtained from all participants. This study received ethical approval from the Joint UCL/UCLH Ethics Committee.

### Biochemical tests

Biochemical tests included follicle-stimulating hormone (FSH), luteinising hormone (LH), oestradiol (E_2_), inhibins A and B, activin A and antimüllerian hormone (AMH). Blood samples (15 ml) were obtained from all subjects during the early follicular phase of the menstrual cycle (day 2–5). Four-timed samples were performed, 15 min apart (to account for the pulsatile variation of FSH release). Following the last-timed sample, the gonadotrophin analogue stimulation test (G-test) was commenced (Ranieri *et al*, 1998). This involved administering a GnRHa – Buserelin 1 mg subcutaneously. This route was chosen to ensure compliance in preference to intranasal administration, which would have involved four divided doses over 24 h. Administration of the G-test was intended to produce a transient ovarian stimulation. This response is quantified after performing a single repeat blood sample approximately 24 h later.

Serum was separated and stored at −20°C before hormone measurements. Assaying each of the four samples and determining an average value then determined mean serum concentrations for FSH, LH and E_2_. For the inhibins and AMH, pooled samples were obtained from the four-timed samples before assaying (obtained by mixing equivalent volumes from each timed sample before freezing).

#### Assays

*Follicle-stimulating hormone and LH:* all samples were assayed in duplicate using a commercial Coat a count solid phase Immunoradiometric assay (DPC, Gwynedd, UK). The sensitivity of the assay is 0.06 mIU ml^−1^ and the intra- and interassay variation was <7%.

*Oestradiol:* All samples were assayed in duplicate using a commercial ELISA kit (IBL Immunobiological Laboratories, Hamburg, Germany) according to the manufacturer's protocol. The sensitivity of the assay was 4.6 pg ml^−1^ and the intra- and interassay variation was <6%.

*Inhibin A:* Serum concentrations of dimeric inhibin A were measured in duplicate 50 *μ*l aliquots as described previously (Muttukrishna *et al*, 1994). The sensitivity of the assay was 2 pg ml^−1^ and the intra- and interassay variations were <6%.

*Inhibin B:* All samples were assayed in duplicate using a commercial ELISA kit (Oxford bio innovations-DSL, Oxford, UK) according to the manufacturer's protocol. The sensitivity of the assay is 10 pg ml^−1^. The intra- and interassay variations were <10%.

*Activin A:* This was measured using a two-site ELISA specific for ‘total’ activin A as described previously (Muttukrishna *et al*, 1996). The detection limit of this assay for human recombinant activin A (Genentech INC, San Francisco, CA, USA) was 50 pg ml^−1^. Intra- and interassay variations were 8.5 and 9.8%, respectively.

*Antimullerian hormone:* All samples were assayed in duplicate using a commercial assay kit according to the manufacturer's sensitised assay protocol (Immunotech, Marseille, France). The sensitivity of the assay was 0.098 ng ml^−1^. The intra- and interassay variations were <15% using an in-house quality control pool.

### Biophysical tests

Ultrasound examination was performed on the same day that blood samples were taken (before ovarian stimulation). Inter-observer variability was minimised by having all ultrasound scans performed by a single investigator (AP). Additional support for performing scans was provided specifically by another investigator (BP) if required. An Acuson 128 XP4 (Acuson USA) with a 2.5–4 mHz-vector probe (transabdominal) and a 5–7.5 mHz EC-7 (transvaginal) probe were used to perform the scans.

### Ultrasound parameters

#### Antral follicle count

Round- or oval-shaped echo-free structures from 2 to 10 mm within the ovaries were considered to be follicles. Total antral follicle count (AFC) was the sum of antral follicles measured from both ovaries.

#### Ovarian volume

Ovarian volume was calculated for each ovary using the prolate ellipsoid formula:

Ovarian volume=D1 × D2 × D3 × *π*/6, where D1, D2 and D3 are maximal perpendicular diameters of the ovary.

#### Mean pulsatility index and mean peak systolic velocity

Ovarian stromal blood flow velocity waveforms were obtained by placing the Doppler gate over the ovarian stroma, which had the highest achievable signals, ensuring that no arteries near the surface of the ovary were measured. Peak systolic velocity and pulsatility indices from both ovaries were then combined to provide the mean pulsatility index and mean peak systolic velocity, respectively.

#### Uterus

Uterine cross-sectional area (cm^2^) and endometrial thickness (mm) were calculated by examining the uterus in the sagittal plane.

### Statistical analyses

A normality test was carried out to assess the distribution of data. Most variables in the cross-sectional data analysis had a gaussian distribution. As such, unpaired Student's *t*-tests were carried out to compare biochemical and biophysical parameters between patients and controls. One-way analysis of variance used to compare baseline parameters only. A *P*-value <0.05 was considered statistically significant. When a significant difference was found, a *post hoc* test (Bonferroni's multiple comparisons) was used to compare differences between individual groups. For non-gaussian distribution, the Mann–Whitney test was employed.

Data in the longitudinal group, which had a normal distribution, were analysed using the paired *t*-test. Non-parametric variables were analysed using the Wilcoxon-signed rank test.

Statistical analyses were performed using GraphPad Prism version 3.00 for Windows (GraphPad Software, San Diego CA, USA) and Statistical Package for Social Sciences (SPSS Inc., Chicago, IL, USA).

## RESULTS

All tests performed were well tolerated, with no adverse effects reported. Results are presented as mean values along with the standard error of the mean (s.e.m.).

### Cross-sectional data

#### Clinical parameters

**Figure 1:**
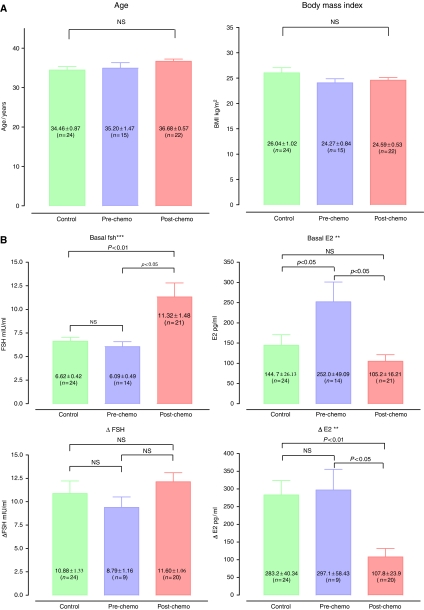

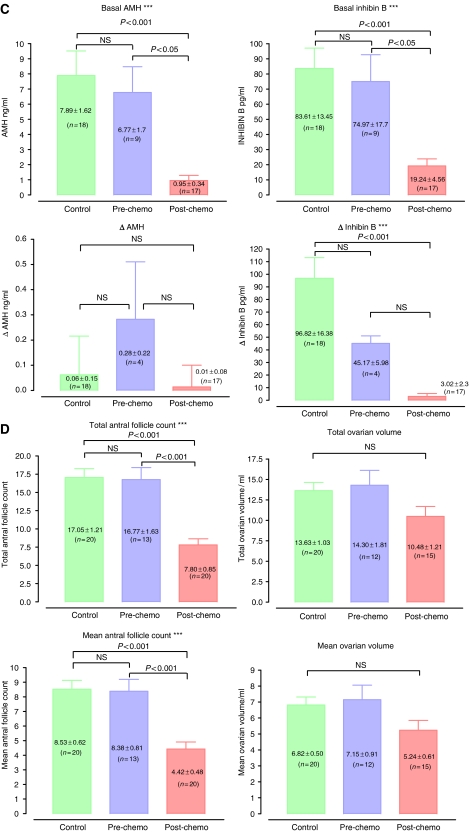
(**A**) Clinical data of study group. This figure illustrates the mean age and BMI, respectively, of patients and controls in the cross-sectional analysis. BMI, body mass index; NS, no statistically significant difference. (**B–D**) Biochemical and biophysical parameters (cross-sectional data). Mean basal hormone parameters are displayed as well as delta (Δ) values, which are obtained by subtracting hormone levels obtained following stimulation from the baseline. (**B**) Follicle-stimulating hormone and E_2_, (**C**) AMH and inhibin B, (**D**) total (and mean) AFC and OV. Significant differences are highlighted with an asterisk (^*^) as follows: ^*^P<0.05; ^**^P<0.01; ^***^P<0.001; NS, no statistically significant difference.

There was no statistically significant difference (*P*>0. 05) in age among all the three groups. The mean age for patients in the pre-chemotherapy group was 35.2±1.5, for patients in the post-chemotherapy group 36.7±0.6 and in the controls 34.5±0.9. Similarly, there was no statistically significant difference in body mass index (BMI) (kg m^−2^) among the three groups. The mean BMI for patients in the pre-chemotherapy group was 24.27±0.84, for patients in the post-chemotherapy group 24.59±0.53 and in the controls 26.04±1.02 (Figure 1A).

#### Biochemical parameters

All biochemical tests included basal (b) estimates as well as stimulated and delta (stimulated – baseline, Δ) results following administration of the G-test. There were no significant differences in biochemical parameters between patients in the pre-chemotherapy arm and the controls.

In the post-chemotherapy arm, there were significant differences when compared to the controls for basal FSH (11.32±1.48 *vs* 6.62±0.42 mIU ml^−1^, *P*<0.001), basal AMH (0.95±0.34 *vs* 7.89±1.62 ng ml^−1^, *P*<0.001) and basal inhibin B (19.24±4.56 *vs* 83.61±13.45 pg ml^−1^, *P*<0.001). One-way ANOVA revealed significant differences in the mean values for all three groups (pre-chemotherapy, post-chemotherapy and controls) for basal FSH, basal AMH and basal inhibin B (*P*<0.001). Basal E_2_ was significantly lower in the control group compared with patients in the pre-chemotherapy group (144.7±26.13 *vs* 252.0± 49.1 pg ml^−1^). Following administration of the G-test, there were significant differences between patients in the post-chemotherapy arm and the controls for stimulated FSH (but not Ä FSH) (22.69± 1.77 *vs* 17.5±1.37 mIU ml^−1^, *P*<0.05); stimulated and delta E_2_ (208.9±37.5 *vs* 427.9±55.87 pg ml^−1^ (stimulated), *P*<0.01; 107.8±23.9 *vs* 283.2±40.34 pg ml^−1^ (delta), *P*< 0.01) and finally stimulated and delta inhibin B (22.5±6.04 *vs* 180.4±22.8 pg ml^−1^ (stimulated), *P*<0.001; 3.02±2.3 *vs* 96.82±16.38 pg ml^−1^ (delta), *P*<0.001). One-way ANOVA revealed significant differences overall among the three groups (pre-chemotherapy, post-chemotherapy and controls) for stimulated FSH (*P*<0.01), stimulated and delta E_2_ (*P*<0.01) and stimulated and delta inhibin B (*P*<0.001) (Figure 1B and C).

#### Biophysical parameters

There were no significant differences in biophysical parameters between patients in the pre-chemotherapy arm and the controls.

In the post-chemotherapy arm, total (and mean) AFC was the only biophysical parameter that displayed a significant difference between patients in the post-chemotherapy arm and the controls (TAFC 7.8±0.9 *vs* 17.1±1.2, *P*<0.001; MAFC 4.4±0.5 *vs* 8.5±0.6, *P*<0.001). One-way ANOVA showed a highly significant difference in the mean TAFC among the three groups (*P*<0.0001). (Figure 1D).

### Longitudinal data

**Figure 2:**
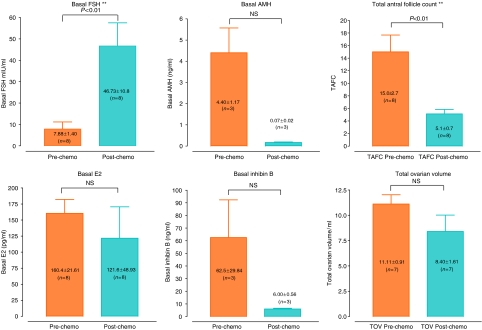
Biochemical and biophysical parameters (longitudinal data). Mean basal levels of FSH, E_2_, AMH, inhibin B, total AFC and total OV are displayed in the same patients tested in the early follicular phase pre-chemotherapy as well as immediately following completion of chemotherapy. Significant differences are highlighted with an asterisk (^*^) as follows: ^*^P< 0.05; ^**^P<0.01; ^***^P<0.001; NS, no statistically significant difference.

In this data set, patients were tested during the early follicular phase of the menstrual cycle preceding chemotherapy and immediately following completion of chemotherapy. It is important to note that patients tested immediately post-chemotherapy were universally amenorrhoeic, and as such were not offered the G-test. All parameters were tested for normality and most were found to have a gaussian distribution. Significant findings in this group are displayed in Figure 2.

#### Biochemical parameters

Basal FSH was significantly lower in patients' pre-chemotherapy compared with post-chemotherapy levels (7.9±1.4 *vs* 46.7±10.8 mIU ml^−1^, *P*<0.01). In addition, basal AMH and basal inhibin B levels were much higher in patients tested pre-chemotherapy compared with post-chemotherapy, but these results were not statistically significant (4.4±1.1 *vs* 0.07±0.02, *P*>0.05 (AMH, ng ml^−1^); 62.5±29.8 *vs* 6.0±0.6, *P*>0.05 (inhibin B, pg ml^−1^)).

#### Biophysical parameters

Both total and mean AFCs were significantly higher in patients tested pre-chemotherapy compared with post-chemotherapy (15.0±2.7 *vs* 5.1±0.7, *P*<0.01 (TAFC); 7.5±1.3 *vs* 2.7±0.3, *P*<0.01 (MAFC). There were no other statistically significant differences between the pre-chemotherapy and post-chemotherapy biophysical markers, including total and mean ovarian volume (OV) (11.1±0.9 *vs* 8.4±1.6, *P*>0.05 (TOV, ml); 5.6±0.5 *vs* 4.3±0.7, *P*>0.05 (MOV, ml).

### Correlations

Correlation statistics were performed using SPSS comparing each of the ovarian reserve markers within each of the three groups studied: controls, pre-chemotherapy and post-chemotherapy patients, respectively. Bivariate analysis was performed using Pearson's correlation coefficients. Significant correlations were flagged at the 0.01 level (two-tailed) or the 0.05 level (two-tailed).

Antimüllerian hormone as a basal marker correlated with ΔE_2_ in patients and the controls (*r*=0.759, *P*<0.001 and *r*=0.518, *P*<0.05, respectively), as well as basal inhibin B (*r*=0.842, *P*<0.001). Furthermore, AMH was the only parameter (in addition to bFSH) to correlate with chronological age in patients only (*r*=0.78, *P*<0.05).

Δ Inhibin B correlated best with biophysical markers of ovarian reserve. In controls, inhibin B correlated positively with TAFC and MAFC (*r*=0.494, *P*<0.05; *r*=0.509, *P*<0.05), whereas Δ E_2_ correlated positively with TAFC in the pre-chemotherapy group, but not the controls (*r*=0.670, *P*<0.05).

Overall, basal markers, which appeared to correlate the most with other markers, were AMH and inhibin B. Post-stimulation, however, E_2_ and inhibin B were the only markers to correlate with AFC. No correlations with OV were identified.

## DISCUSSION

The results of this study show that biochemical and biophysical tests may be used to accurately estimate ovarian reserve in premenopausal recipients of chemotherapy for breast cancer.

This study confirms the observation of a recent paper where the new ovarian reserve tests, such as inhibin B, AMH and AFC, were investigated in breast cancer patients before and after treatment (Anderson *et al*, 2006). However, our study is the first in which dynamic ovarian reserve testing (G-test) has been evaluated in breast cancer patients. In an IVF setting, basal ovarian markers can be normal yet these patients do not respond to stimulation. Hence, dynamic tests are used in the assisted reproductive technology setting as they give a better indication of the ovarian response to stimulation. We believe the G-test can add to the discriminatory capacity provided by basal markers alone, as the underlying pathophysiology of chemotherapy-mediated gonadotoxicity is in fact dynamic.

In the recently published study, AMH concentration in particular was found to be useful as an early indicator of ovarian ageing, including the assessment of chemotherapy-induced ovarian follicle loss (Anderson *et al*, 2006). Contrary to this study where the patients were 28–52 years of age, our study cohort comprised a significantly younger patient cohort (mean age of patients was 41.3 in the chemotherapy arm of the Anderson study, compared with 35.2 and 36.7 in the pre-chemotherapy and post-chemotherapy arms of our study), and included an age-matched control group with proven fertility. This was in keeping with our objective, which was to evaluate ORT in young women with breast cancer. It is this group where validation of ORT could potentially lead to adequate counselling and treatment with regards fertility preservation, at a time when therapeutic intervention is still feasible.

Previous studies in breast cancer were focused mainly on the incidence of amenorrhoea and premature menopause (Bines *et al*, 1996; Goodwin *et al*, 1999). Other investigators assessed the endocrine consequences of chemotherapy in patients with breast cancer, but the analysis was limited to gonadotrophin and steroid alterations (Padmanabhan *et al*, 1987; Dowsett and Richner, 1991; Mehta *et al*, 1992). These studies were performed long before the concept of ovarian reserve was realised. The importance of these alterations were accurately surmised as being detrimental to fertility, but the limitations of these studies prevented meaningful application in clinical practice (Hensley and Reichman, 1998). Prospective studies are limited and have been performed in women and men with haematological malignancy (Chatterjee *et al*, 1994; Wallace *et al*, 1997).

Our data suggest that ovarian reserve was intact before chemotherapy in a breast cancer cohort, implying that multi-agent chemotherapy was the main factor that led to diminished ovarian reserve. Alkylating agents such as cyclophosphamide, which are non cell-cycle specific, are more toxic to the ovaries than cell-cycle-specific agents, such as methotrexate and fluorouracil. It is unclear, however, what effect multi-agent chemotherapy has on ovarian reserve, especially, given the fact that newer agents are continually being developed (Awada *et al*, 2003). All the biochemical and biophysical markers with the exception of basal oestradiol (bE_2_) levels provided evidence that ovarian reserve was intact before chemotherapy.

The G-test is a dynamic ovarian reserve test. It was chosen in this study, as it provided an ovarian response, which was less potent than the HMG test, an important factor to consider in patients with breast cancer. Although bE_2_ levels were significantly higher in patients (pre-chemotherapy) compared with the controls, there was no significant difference between stimulated and delta E_2_ levels. This may be explained by the fact that bE_2_ levels correlate more with the underlying disease, as it has been shown that high plasma E_2_ concentrations have been linked to breast cancer development regardless of menopausal status (Clamp *et al*, 2002). Oestradiol increase in response to the G-test, however, probably correlates better with ovarian reserve, which would explain the lack of a significant difference in patients compared with the controls in addition to the other markers of ovarian reserve.

Perhaps, the main factor involved in assessing these patients appropriately is the fact that the pathophysiological process is dynamic, with both acute and cumulative effects. As such, efforts to grade the extent of ovarian damage, and indeed ovarian recovery by clinical, biochemical and biophysical parameters are limited by the dynamic processes involved (Chatterjee and Kottaridis, 2002). Furthermore, extrapolation of data from one cancer cohort to another is inappropriate due to differences in disease, type of chemotherapy and the demographics of the population studied.

In the fertility setting, the most important aspect of diminished ovarian reserve and the associated decline in reproductive potential is that its onset is highly variable (Scott and Hofmann, 1995). To the best of our knowledge, it appears that no single marker can be clinically useful in assessing ovarian reserve, leading to the evaluation of multiple markers (Lutchman Singh *et al*, 2005).

Our study group comprised regularly cycling patients and controls. Although all patients had undergone a variable period of amenorrhoea following chemotherapy, clinical characteristics alone (patient age, BMI and parity) were not found to be discriminatory once cyclical activity resumed. This is in keeping with the generally accepted notion that clinical characteristics are not reliable estimates of reproductive age.

Biochemical parameters, which appeared to discriminate between patients and the controls in the cross-sectional data set, were serum FSH, E_2_, inhibin B and AMH. The data relating to the use of FSH in estimating ovarian reserve in breast cancer are limited. Although widely used in a reproductive medicine setting, its main limitations arise from a lack of reproducibility (Sharara *et al*, 1998). The pulsatile nature of FSH secretion is believed to be one of the factors responsible for this variation. To minimise this possibility, we took four (4)-timed samples, 15 min apart and used a mean value as a ‘true’ representative figure. Basal FSH levels were significantly different across the three groups (controls, pre-chemotherapy and post-chemotherapy patients, *P*=0.0006). Following the G-test, stimulated FSH levels were also significantly different (*P*=0.01). However, ΔFSH levels did not confer any discriminatory capacity. Follicle-stimulating hormone is an indirect marker of ovarian reserve and depends on the presence of an intact hypothalamic-pituitary-ovarian (HPO) axis. Administration of the G-test causes a temporary increase in pituitary secretion of FSH and LH, to which the ovaries respond by releasing E_2_. It is the amount of E_2_ released (Δ), which correlates with ovarian reserve, not ΔFSH, which is in keeping with the results.

Basal oestradiol levels were significantly higher in the pre-chemotherapy group compared with the controls. This may have a role in the pathophysiological process of breast cancer as described earlier. No significant difference existed between the controls and the post-chemotherapy group. In fact, higher levels of bE_2_ were seen in the pre-chemotherapy group as opposed to the post-chemotherapy, which one would not expect, as higher levels of E_2_ are associated with diminished ovarian reserve. Both stimulated and delta E_2_ levels were significantly different, however, being much lower in the post-chemotherapy group compared with the pre-chemotherapy group and the controls, respectively. This supports the notion that the G-test confers discriminatory capacity.

Basal, stimulated and Δ inhibin B levels were all significantly different between patients and the controls. Although inhibin B is mainly secreted by pre-antral follicles (Klein *et al*, 1996), inhibin A is produced primarily during the late follicular phase by mature follicles and the corpus luteum (Roberts *et al*, 1993). This would explain why the former is a more reliable marker of ovarian reserve, as our results confirm. The added discriminatory capacity as provided by the G-test for inhibin B and E_2_ is consistent with the theory that the pathophysiological processes taking place within the ovary are in fact dynamic – thus necessitating a dynamic form of assessment.

Antimullerian hormone is considered a direct marker of ovarian reserve, as it is produced by FSH-sensitive early antral follicles. In this way, it may be a more sensitive predictor of ovarian reserve than other markers, such as AFC and inhibin B, which detect more mature primordial follicles. Antimullerian hormone has a relatively stable expression over the menstrual cycle (Cook *et al*, 2000; La Marca *et al*, 2006). This would explain why serum AMH levels were mostly unchanged in our study following the G-test, a phenomenon detected in other studies (van Rooij *et al*, 2002; Pastor *et al*, 2005). Our results confirm that basal AMH levels were dramatically reduced in the post-chemotherapy group compared with both the controls and the pre-chemotherapy group.

With regard to biophysical parameters, our data suggest that AFC is most useful. Although studies in cancer are limited, AFC and OV have been found to be useful in assessing ovarian reserve in childhood survivors of cancer (Larsen *et al*, 2003a, 2003b) and patients undergoing bone marrow transplantation (Chatterjee *et al*, 1994). Our results did not show a significant difference in OV between patients and the controls, although it was approaching significance (*P*=0.06).

Longitudinal data analysis revealed that the parameters, which were discriminatory between these two groups, were FSH, E_2_ and AFC. It can be deduced that the multi-agent chemotherapy received by these patients suppressed ovarian function resulting in a hypoestrogenic state, with a corresponding high FSH and amenorrhoea. Reduced AFC reflects a reduction in the number of antral follicles and is consistent with our observation in the cross-sectional population (post-chemotherapy cycling women). Ovarian volume, as was the case in the cross-sectional data, was not significantly different between both groups. This may be a reflection of the fact that the induced hypoestrogenic state was temporary. It can be argued that in terms of reproductive ageing, a reduction in OV is a relatively late event that comes about after a protracted period of diminished ovarian reserve. It is plausible that despite these patients being amenorrhoeic, the unchanged OVs implied that resumption of ovarian function, albeit at a level lower than the pre-chemotherapy state, was likely to occur. In fact, all patients in the post-chemotherapy arm of the cross-sectional analysis had resumed normal menses, indicating that the effects of the multi-agent chemotherapy received were at least partially reversible.

In summary, this study confirms the use of biochemical and biophysical parameters of ovarian reserve in a breast cancer cohort. The methodology was robust and included elaborate methods of testing – all of which appeared to be well tolerated. The cohort was disease standardised and received cyclophosphamide-based chemotherapy. Biochemical markers, which appeared to be discriminatory, included FSH, AMH, inhibin B and E_2_ in response to the G-test. The only clearly discriminatory biophysical marker was the AFC. There was good correlation between AFC and other markers of ovarian reserve. These results taken together support the hypothesis that ovarian reserve is reduced in young, regularly cycling women following treatment with chemotherapeutic agents for breast cancer. The data also add to the understanding of the pathophysiological processes involved.

These findings have major implications for breast cancer survivors, for whom reproductive issues are a major concern (Partridge *et al*, 2004). Furthermore, the potential exists for ovarian reserve testing to be applied in patients with different types of cancer. To achieve this, a large sample size should be followed up longitudinally to determine the potential therapeutic role of ovarian reserve testing in this cohort.

In conclusion, this study confirms that ovarian reserve can be assessed in breast cancer patients using AFC, inhibin B and AMH to inform patients on their prospects of fertility treatment post chemotherapy.

## Figures and Tables

**Table 1:** Clinical data of patients in longitudinal group

**PT.**	**Age/ years**	**Parity**	**Cycle/ days**	**BMI (kg/m^2^)**	**Histological diagnosis**	**Chemotherapy regime**	**Cumulative dose**
1	39	0+0	5/32	25	23 mm grade 3 ductal ca; ER/PR moderately pos, HER2 3+0/7 nodes pos	FEC × 6	F=5760 mg E=576 mg C=5760 mg
2	35	0+0	7/30	23	50 mm grade 2/3 ductal ca; ER weakly pos, 2/18 nodes pos	FEC × 6	F=6600 mg E=660 mg C=6600 mg
3	32	0+0	4/26	24	18 mm grade 3 ductal ca, weakly ER pos 1/14 nodes pos	EC × 4 Paclitaxel × 4 Gemcitabine × 4	E=688 mg C=4400 mg P=1392 mg G=19152 mg
4	35	0+0	5/30	30	25 mm grade 2 ductal ca; ER/PR neg; HER2 3 pos; 0/8 nodes pos	FEC × 6	F=6480 mg E=648 mg C=6480 mg
5	33	0+0	5/30	21	grade 3 ductal ca, ER/PR neg, HER2 1pos	FEC × 6	F=5810 mg E=596 mg C=5810 mg
6	41	1+1	5/28	26	42mm high grade DCIS with 2 invasive foci grade 2; ER/PR neg; 2/12 nodes pos	EC × 4 paclitaxelx4	E=702 mg C=4700 mg P=1368 mg herceptin
7	31	0+1	3/34	20	Grade 3 Invasive ductal ca; ER/PR neg; HER2 3pos	EC × 4 paclitaxelx4	E=564 mg C=3740 mg P=1074 mg herceptin
8	38	0+0	6/28	25	Grade 2 ductal carcinoma, ERpos, HER2neg	E × 4 CMF × 4	E=8284 mg C=9920 mg M=640 mg F=9600 mg

BMI, body mass index; C, cyclophosphamide; E, epirubicin; ER, oestrogen receptor status; F, fluorouracil; G, gemcitabine; HER2, HER2 receptor status; M, methotrexate; neg, negative; P, paclitaxel; pos, positive; PR, progesterone receptor status.

The patients in this group comprised the longitudinal arm of the study, with all patients having ovarian reserve tests performed before receiving chemotherapy. Cycle length is depicted first by menstruation length followed by cycle length in days. As per the study protocol, all patients in this group had ovarian reserve tests performed between cycle days 2–5. Relevant details of diagnosis and chemotherapy regimen (including cumulative dosages) are shown.
